# Ethyl 8-meth­oxy-4-oxo-1,4-di­hydro­quinoline-3-carboxyl­ate

**DOI:** 10.1107/S1600536814011854

**Published:** 2014-05-31

**Authors:** Yoshinobu Ishikawa, Nanako Yoshida

**Affiliations:** a School of Pharmaceutical Sciences, University of Shizuoka, 52-1 Yada, Suruga-ku, Shizuoka 422-8526, Japan

## Abstract

In the title compound, C_13_H_13_NO_4_, the asymmetric unit contains four independent mol­ecules, each exhibiting an intra­molecular N—H⋯O hydrogen bond. The ethyl group in one of the four mol­ecules is disordered, with a refined occupancy ratio of 0.295 (16):0.705 (16). A face-to-face stacking inter­action is found between the benzene rings of the quinoline units of two of the mol­ecules [centroid–centroid distance = 3.541 (2) Å], which are sandwiched by the other two mol­ecules through N—H⋯O hydrogen bonding. In the crystal, the sandwiched mol­ecules are assembled *via* stacking inter­actions along the *b*-axis direction with their translation-symmetry equivalents [centroid–centroid distance = 3.529 (2) Å], and are further linked through N—H⋯O hydrogen bonding. The other two mol­ecules are linked *via* stacking inter­actions with their inversion-symmetry equivalents [centroid–centroid distances = 3.512 (3) and 3.716 (4) Å] and *via* N—H⋯O hydrogen bonding.

## Related literature   

For the background of this study, see: Ishikawa & Fujii (2011 ▶). For the synthesis of the title compound, see: Ozeki *et al.* (1987 ▶).
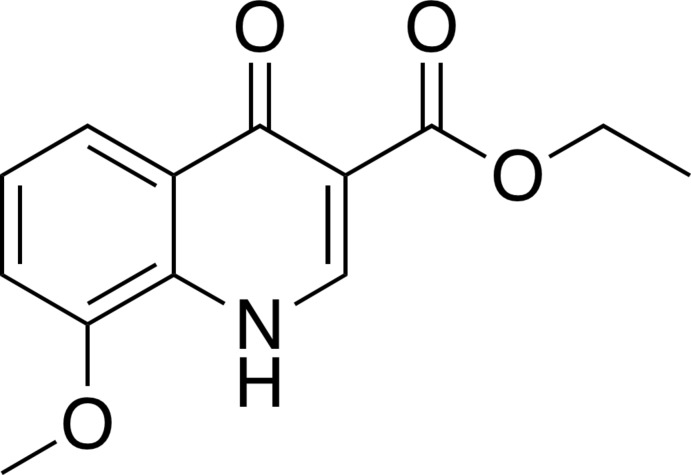


## Experimental   

### 

#### Crystal data  

C_13_H_13_NO_4_
*M* *_r_* = 247.25
Monoclinic, 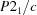

*a* = 26.32 (3) Å
*b* = 7.0628 (15) Å
*c* = 25.305 (16) Å
β = 102.24 (6)°
*V* = 4598 (5) Å^3^
*Z* = 16
Mo *K*α radiation
μ = 0.11 mm^−1^
*T* = 100 K
0.40 × 0.23 × 0.13 mm

#### Data collection  

Rigaku AFC-7R diffractometer
12488 measured reflections
10546 independent reflections
5412 reflections with *F* ^2^ > 2σ(*F* ^2^)
*R*_int_ = 0.040
3 standard reflections every 150 reflections intensity decay: 6.7%

#### Refinement  

*R*[*F* ^2^ > 2σ(*F* ^2^)] = 0.053
*wR*(*F* ^2^) = 0.130
*S* = 0.99
10546 reflections
677 parameters
H-atom parameters constrained
Δρ_max_ = 0.32 e Å^−3^
Δρ_min_ = −0.31 e Å^−3^

### 

Data collection: *WinAFC* (Rigaku, 1999 ▶); cell refinement: *WinAFC*; data reduction: *WinAFC*; program(s) used to solve structure: *SIR2008* (Burla, *et al.*, 2007 ▶); program(s) used to refine structure: *SHELXL97* (Sheldrick, 2008 ▶); molecular graphics: *CrystalStructure* (Rigaku, 2010 ▶); software used to prepare material for publication: *CrystalStructure*.

## Supplementary Material

Crystal structure: contains datablock(s) General, I. DOI: 10.1107/S1600536814011854/rn2125sup1.cif

Structure factors: contains datablock(s) I. DOI: 10.1107/S1600536814011854/rn2125Isup2.hkl

Supporting information file. DOI: 10.1107/S1600536814011854/rn2125Isup3.cml

CCDC reference: 1004530

Additional supporting information:  crystallographic information; 3D view; checkCIF report

## Figures and Tables

**Table 1:** Hydrogen-bond geometry (Å, °)

*D*—H⋯*A*	*D*—H	H⋯*A*	*D*⋯*A*	*D*—H⋯*A*
N1—H1*A*⋯O2	0.88	2.33	2.665 (3)	103
N1—H1*A*⋯O5	0.88	1.93	2.729 (3)	151
N2—H2⋯O3^i^	0.88	2.19	2.873 (3)	134
N2—H2⋯O6	0.88	2.31	2.656 (4)	103
N3—H3⋯O10	0.88	2.35	2.685 (3)	103
N3—H3⋯O13^ii^	0.88	2.21	2.857 (4)	130
N3—H3⋯O15^ii^	0.88	2.26	2.898 (3)	129
N4—H4*A*⋯O9	0.88	2.03	2.716 (3)	134
N4—H4*A*⋯O14	0.88	2.31	2.652 (3)	103

Symmetry codes: (i) 
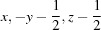
; (ii) 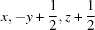.

**Fig. 1.**
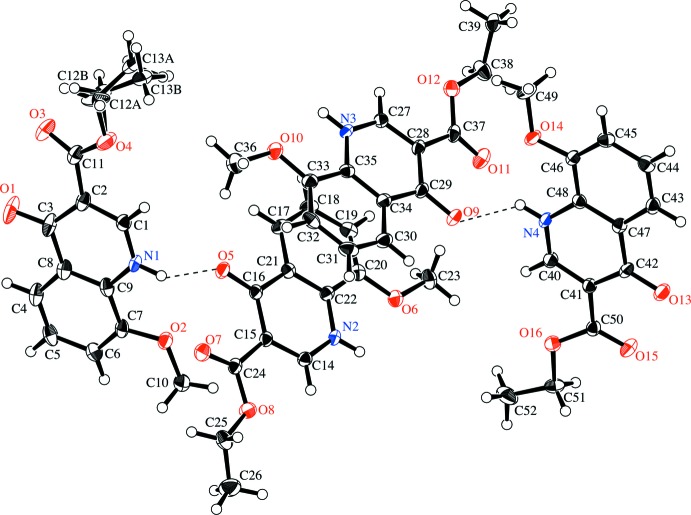
The molecular structure of the title compound, with displacement ellipsoids drawn at the 50% probability level. Hydrogen atoms are shown as small spheres of arbitrary radii. Intermolecular hydrogen bonds are represented as dashed lines.

**Fig. 2.**
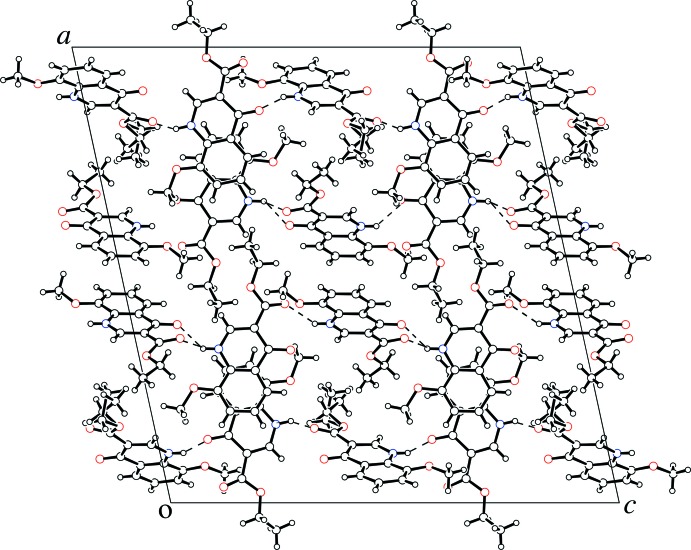
A crystal packing view of the title compound. Intermolecular hydrogen bonds are represented as dashed lines.

## supplementary crystallographic information

### 1. Comment

4-Quinolones show inhibition not only to Gram negative and Gram positive bacteria, but also to human immunodeficiency virus (HIV). The inhibition to HIV is derived from their chelating ability to metal ions in the active site of the metalloenzyme HIV integrase. According to our inhibitor design targeting the metalloenzyme influenza virus RNA polymerase (Ishikawa & Fujii, 2011), we synthesized the title compound as an intermediate of final products.

The asymmetric unit contains four independent molecules with intramolecular N–H···O hydrogen bonding, and the ethyl group in one of the four molecules is disordered with a refined occupancy ratio of 0.295 (16):0.705 (16), as shown in Fig.1. A face-to-face stacking interaction is found between the benzene rings of the quinoline units of the two molecules [centroid–centroid distances = 3.541 (2) Å], which are sandwiched by the other two molecules through intermolecular N–H···O hydrogen bonding.

In the crystal, the sandwiched molecules are assembled *via* stacking interaction along the *b*-axis direction with the translation-symmetry equivalents^i,ii^ [centroid-centroid distances between the benzene rings of the quinoline units = 3.529 (2) Å, i: *x*, *y* + 1, *z*, ii: *x*, *y*–1, *z*], and are further linked with the glide-reflection-symmetry equivalents through N–H···O hydrogen bonding. The other two molecules are also linked with the inversion-symmetry equivalents^iii,iv^*via* stacking interactions [centroid-centroid distances between the benzene rings of the quinoline units = 3.512 (3) and 3.716 (4) Å, iii: –*x* + 1, –*y* + 1, –*z*, iv: –*x* + 2, –*y*–1, –*z* + 1], and with the glide-reflection-symmetry equivalents *via* N–H···O hydrogen bonding, as shown in Fig.2. A l l 1,3-diketones and secondary amines of the molecules are involved in the intermolecular hydrogen bonding, which leads to a higher order network.

### 2. Experimental

The title compound was synthesized according to the literature (Ozeki *et al*. 1987). Single crystals suitable for X-ray diffraction were obtained by slow evaporation of an acetonitrile solution of the compound at room temperature.

### 3. Refinement

The hydrogen atoms of secondary amine [N–H 0.88 Å, *U*_iso_(H) = 1.2*U*_eq_(N)], methylene [C–H = 0.99 Å, *U*_iso_(H) = 1.2*U*_eq_(C)], and phenyl groups [C–H 0.95 Å, *U*_iso_(H) = 1.2*U*_eq_(C)] were placed in geometrical positions, and refined using a riding model. A rotating group model was applied with a distance constraint for the hydrogen atoms of methyl groups [C–H = 0.98 Å, *U*_iso_(H) = 1.2*U*_eq_(C)]. The ethyl group was disordered and the relative occupancies of the two orientations were refined to 0.295 (16) and 0.705 (16).

### Crystal data

**Table:**

C_13_H_13_NO_4_	*F*(000) = 2080.00
*M**_r_* = 247.25	*D*_x_ = 1.429 Mg m^−^^3^
Monoclinic, *P*2_1_/*c*	Mo *K*α radiation, λ = 0.71069 Å
Hall symbol: -P 2ybc	Cell parameters from 4 reflections
*a* = 26.32 (3) Å	θ = 16.5–16.7°
*b* = 7.0628 (15) Å	µ = 0.11 mm^−^^1^
*c* = 25.305 (16) Å	*T* = 100 K
β = 102.24 (6)°	Plate, colorless
*V* = 4598 (5) Å^3^	0.40 × 0.23 × 0.13 mm
*Z* = 16	

### Data collection

**Table:**

Rigaku AFC-7R diffractometer	θ_max_ = 27.5°
ω scans	*h* = −19→34
12488 measured reflections	*k* = 0→9
10546 independent reflections	*l* = −32→32
5412 reflections with *F*^2^ > 2σ(*F*^2^)	3 standard reflections every 150 reflections
*R*_int_ = 0.040	intensity decay: 6.7%

### Refinement

**Table:**

Refinement on *F*^2^	Secondary atom site location: difference Fourier map
*R*[*F*^2^ > 2σ(*F*^2^)] = 0.053	Hydrogen site location: inferred from neighbouring sites
*wR*(*F*^2^) = 0.130	H-atom parameters constrained
*S* = 0.99	*w* = 1/[σ^2^(*F*_o_^2^) + (0.0467*P*)^2^] where *P* = (*F*_o_^2^ + 2*F*_c_^2^)/3
10546 reflections	(Δ/σ)_max_ < 0.001
677 parameters	Δρ_max_ = 0.32 e Å^−^^3^
0 restraints	Δρ_min_ = −0.31 e Å^−^^3^
Primary atom site location: structure-invariant direct methods	

### Special details

**Table:**

Refinement. Refinement was performed using all reflections. The weighted *R*-factor (*wR*) and goodness of fit (*S*) are based on *F*^2^. *R*-factor (gt) are based on *F*. The threshold expression of *F*^2^ > 2.0 σ(*F*^2^) is used only for calculating *R*-factor (gt).

### Fractional atomic coordinates and isotropic or equivalent isotropic displacement parameters (Å^2^)

**Table:**

	*x*	*y*	*z*	*U*_iso_*/*U*_eq_	Occ. (<1)
O1	0.90402 (9)	−0.3789 (3)	0.63683 (7)	0.0463 (6)	
O2	0.91957 (6)	−0.4270 (3)	0.40622 (6)	0.0248 (5)	
O3	0.83651 (8)	−0.0767 (3)	0.64383 (7)	0.0383 (6)	
O4	0.83284 (8)	0.1380 (3)	0.57824 (7)	0.0376 (5)	
O5	0.86082 (7)	−0.0095 (3)	0.38683 (6)	0.0252 (5)	
O6	0.73179 (6)	−0.2342 (3)	0.17433 (6)	0.0258 (5)	
O7	0.96521 (7)	0.0186 (3)	0.37428 (7)	0.0309 (5)	
O8	0.97691 (6)	−0.0932 (3)	0.29473 (6)	0.0270 (5)	
O9	0.66478 (7)	0.2109 (3)	0.15921 (6)	0.0290 (5)	
O10	0.74812 (6)	0.3555 (3)	0.39298 (6)	0.0277 (5)	
O11	0.56330 (7)	0.0661 (3)	0.14972 (7)	0.0307 (5)	
O12	0.52678 (6)	0.1701 (3)	0.21704 (6)	0.0260 (5)	
O13	0.60894 (7)	0.3565 (3)	−0.10036 (6)	0.0287 (5)	
O14	0.56294 (7)	0.4991 (3)	0.11598 (6)	0.0256 (5)	
O15	0.63913 (7)	−0.0263 (3)	−0.10040 (6)	0.0279 (5)	
O16	0.67734 (6)	−0.1264 (3)	−0.01723 (6)	0.0253 (5)	
N1	0.89152 (8)	−0.2074 (3)	0.48081 (8)	0.0209 (5)	
N2	0.82785 (7)	−0.1588 (3)	0.22772 (7)	0.0188 (5)	
N3	0.66231 (7)	0.2686 (3)	0.31878 (7)	0.0199 (5)	
N4	0.60684 (7)	0.2617 (3)	0.05784 (8)	0.0212 (5)	
C1	0.87218 (9)	−0.0994 (4)	0.51514 (9)	0.0222 (6)	
C2	0.87279 (10)	−0.1523 (4)	0.56782 (9)	0.0237 (6)	
C3	0.89838 (11)	−0.3262 (4)	0.58935 (10)	0.0297 (7)	
C4	0.94008 (10)	−0.6225 (4)	0.56378 (10)	0.0293 (7)	
C5	0.95624 (10)	−0.7339 (4)	0.52657 (10)	0.0289 (7)	
C6	0.94985 (9)	−0.6756 (4)	0.47227 (10)	0.0256 (7)	
C7	0.92876 (9)	−0.5015 (4)	0.45748 (9)	0.0227 (6)	
C8	0.91780 (10)	−0.4447 (4)	0.54940 (10)	0.0250 (6)	
C9	0.91301 (9)	−0.3833 (4)	0.49606 (10)	0.0228 (6)	
C10	0.93615 (10)	−0.5370 (4)	0.36561 (10)	0.0284 (7)	
C11	0.84653 (10)	−0.0316 (5)	0.60098 (10)	0.0267 (7)	
C14	0.87688 (9)	−0.1256 (4)	0.25245 (9)	0.0194 (6)	
C15	0.89141 (9)	−0.0763 (4)	0.30612 (9)	0.0185 (6)	
C16	0.85213 (9)	−0.0599 (4)	0.33851 (9)	0.0186 (6)	
C17	0.75829 (10)	−0.1170 (4)	0.33859 (10)	0.0221 (6)	
C18	0.70937 (10)	−0.1697 (4)	0.31279 (10)	0.0252 (7)	
C19	0.69827 (10)	−0.2100 (4)	0.25725 (10)	0.0228 (6)	
C20	0.73699 (9)	−0.2008 (4)	0.22867 (9)	0.0211 (6)	
C21	0.79901 (9)	−0.1100 (4)	0.31026 (9)	0.0193 (6)	
C22	0.78789 (9)	−0.1553 (4)	0.25547 (9)	0.0187 (6)	
C23	0.68089 (10)	−0.2780 (5)	0.14411 (10)	0.0292 (7)	
C24	0.94706 (9)	−0.0435 (4)	0.32993 (9)	0.0199 (6)	
C25	1.03194 (9)	−0.0446 (5)	0.30958 (10)	0.0284 (7)	
C26	1.05949 (10)	−0.1754 (5)	0.27812 (11)	0.0424 (9)	
C27	0.61936 (9)	0.2219 (4)	0.28373 (9)	0.0194 (6)	
C28	0.61673 (9)	0.1997 (4)	0.22912 (9)	0.0191 (6)	
C29	0.66297 (10)	0.2287 (4)	0.20787 (9)	0.0209 (6)	
C30	0.75633 (10)	0.3259 (4)	0.23116 (10)	0.0255 (7)	
C31	0.79947 (10)	0.3795 (4)	0.26847 (11)	0.0283 (7)	
C32	0.79854 (10)	0.3938 (4)	0.32319 (10)	0.0244 (6)	
C33	0.75342 (10)	0.3531 (4)	0.34052 (10)	0.0220 (6)	
C34	0.70964 (9)	0.2869 (4)	0.24745 (9)	0.0201 (6)	
C35	0.70828 (9)	0.3033 (4)	0.30219 (9)	0.0186 (6)	
C36	0.79478 (10)	0.3907 (5)	0.43267 (10)	0.0391 (8)	
C37	0.56764 (10)	0.1390 (4)	0.19392 (9)	0.0216 (6)	
C38	0.47610 (9)	0.1025 (4)	0.18866 (11)	0.0307 (7)	
C39	0.43571 (10)	0.2332 (5)	0.20133 (10)	0.0315 (7)	
C40	0.62723 (9)	0.1348 (4)	0.03059 (9)	0.0217 (6)	
C41	0.62767 (9)	0.1521 (4)	−0.02366 (9)	0.0193 (6)	
C42	0.60759 (9)	0.3223 (4)	−0.05229 (9)	0.0202 (6)	
C43	0.56329 (9)	0.6329 (4)	−0.04349 (10)	0.0225 (6)	
C44	0.54174 (9)	0.7582 (4)	−0.01378 (10)	0.0246 (6)	
C45	0.54136 (9)	0.7219 (4)	0.04069 (10)	0.0232 (6)	
C46	0.56207 (9)	0.5571 (4)	0.06416 (9)	0.0198 (6)	
C47	0.58544 (9)	0.4628 (4)	−0.02048 (9)	0.0190 (6)	
C48	0.58500 (9)	0.4274 (4)	0.03387 (9)	0.0186 (6)	
C49	0.53443 (11)	0.6118 (4)	0.14692 (10)	0.0306 (7)	
C50	0.64771 (9)	−0.0045 (4)	−0.05171 (9)	0.0207 (6)	
C51	0.69833 (10)	−0.2836 (4)	−0.04280 (10)	0.0278 (7)	
C52	0.72593 (11)	−0.4115 (5)	0.00148 (12)	0.0383 (8)	
H1A	0.8906	−0.1660	0.4478	0.0251*	
H1	0.8573	0.0193	0.5028	0.0266*	
H2	0.8207	−0.1836	0.1928	0.0225*	
H3	0.6617	0.2778	0.3533	0.0238*	
H4A	0.6070	0.2411	0.0922	0.0254*	
H4	0.9440	−0.6660	0.5999	0.0351*	
H5	0.9720	−0.8526	0.5374	0.0347*	
H6	0.9601	−0.7561	0.4463	0.0307*	
H10A	0.9183	−0.6596	0.3621	0.0340*	
H10B	0.9738	−0.5571	0.3759	0.0340*	
H10C	0.9277	−0.4697	0.3310	0.0340*	
H14	0.9032	−0.1364	0.2320	0.0233*	
H17	0.7649	−0.0848	0.3759	0.0265*	
H18	0.6826	−0.1790	0.3326	0.0303*	
H19	0.6640	−0.2437	0.2395	0.0274*	
H23A	0.6819	−0.2930	0.1059	0.0351*	
H23B	0.6570	−0.1750	0.1480	0.0351*	
H23C	0.6688	−0.3960	0.1577	0.0351*	
H25A	1.0452	−0.0620	0.3489	0.0341*	
H25B	1.0374	0.0890	0.3003	0.0341*	
H26A	1.0441	−0.1637	0.2395	0.0508*	
H26B	1.0559	−0.3062	0.2897	0.0508*	
H26C	1.0964	−0.1416	0.2847	0.0508*	
H27	0.5886	0.2025	0.2969	0.0233*	
H30	0.7580	0.3151	0.1941	0.0306*	
H31	0.8307	0.4074	0.2569	0.0339*	
H32	0.8289	0.4315	0.3485	0.0292*	
H36A	0.8069	0.5198	0.4283	0.0469*	
H36B	0.8216	0.3000	0.4279	0.0469*	
H36C	0.7877	0.3764	0.4689	0.0469*	
H38A	0.4701	−0.0277	0.2005	0.0369*	
H38B	0.4744	0.1004	0.1492	0.0369*	
H39A	0.4406	0.2468	0.2406	0.0378*	
H39B	0.4011	0.1811	0.1866	0.0378*	
H39C	0.4390	0.3573	0.1851	0.0378*	
H40	0.6425	0.0252	0.0492	0.0260*	
H43	0.5634	0.6607	−0.0802	0.0270*	
H44	0.5267	0.8719	−0.0301	0.0296*	
H45	0.5268	0.8117	0.0612	0.0278*	
H49A	0.4977	0.6149	0.1287	0.0368*	
H49B	0.5483	0.7410	0.1502	0.0368*	
H49C	0.5378	0.5566	0.1830	0.0368*	
H51A	0.7229	−0.2367	−0.0645	0.0334*	
H51B	0.6700	−0.3530	−0.0670	0.0334*	
H52A	0.7009	−0.4613	0.0217	0.0459*	
H52B	0.7530	−0.3398	0.0260	0.0459*	
H52C	0.7419	−0.5168	−0.0143	0.0459*	
C12A	0.8186 (9)	0.296 (4)	0.6135 (10)	0.044 (6)	0.295 (16)
C12B	0.8007 (4)	0.2488 (15)	0.6076 (4)	0.0358 (19)	0.705 (16)
C13B	0.7774 (3)	0.4098 (10)	0.5748 (3)	0.0350 (18)	0.705 (16)
C13A	0.7592 (9)	0.319 (4)	0.5888 (5)	0.059 (7)	0.295 (16)
H12A	0.8381	0.4133	0.6101	0.0524*	0.295 (16)
H12B	0.8249	0.2575	0.6520	0.0524*	0.295 (16)
H13A	0.7543	0.3575	0.5508	0.0705*	0.295 (16)
H13B	0.7447	0.4168	0.6090	0.0705*	0.295 (16)
H13C	0.7413	0.1989	0.5912	0.0705*	0.295 (16)
H12C	0.7729	0.1675	0.6163	0.0429*	0.705 (16)
H12D	0.8224	0.2951	0.6420	0.0429*	0.705 (16)
H13D	0.8042	0.4749	0.5599	0.0420*	0.705 (16)
H13E	0.7625	0.4980	0.5973	0.0420*	0.705 (16)
H13F	0.7499	0.3645	0.5451	0.0420*	0.705 (16)

### Atomic displacement parameters (Å^2^)

**Table:**

	*U*^11^	*U*^22^	*U*^33^	*U*^12^	*U*^13^	*U*^23^
O1	0.0862 (17)	0.0341 (13)	0.0158 (10)	0.0014 (12)	0.0051 (10)	0.0065 (10)
O2	0.0305 (10)	0.0246 (11)	0.0182 (9)	0.0022 (9)	0.0032 (8)	−0.0007 (9)
O3	0.0503 (13)	0.0461 (14)	0.0220 (10)	−0.0103 (11)	0.0154 (9)	0.0016 (10)
O4	0.0539 (13)	0.0372 (14)	0.0270 (11)	0.0070 (11)	0.0209 (10)	0.0015 (10)
O5	0.0334 (11)	0.0273 (12)	0.0147 (9)	0.0058 (9)	0.0045 (8)	−0.0009 (8)
O6	0.0216 (10)	0.0339 (12)	0.0203 (9)	−0.0003 (9)	0.0012 (8)	−0.0023 (9)
O7	0.0293 (11)	0.0387 (13)	0.0221 (10)	0.0039 (10)	−0.0005 (8)	−0.0099 (10)
O8	0.0202 (9)	0.0379 (13)	0.0229 (9)	−0.0034 (9)	0.0042 (8)	−0.0059 (9)
O9	0.0347 (11)	0.0388 (13)	0.0149 (9)	0.0070 (10)	0.0085 (8)	0.0024 (9)
O10	0.0232 (10)	0.0390 (12)	0.0193 (9)	−0.0031 (9)	0.0011 (8)	−0.0078 (9)
O11	0.0412 (12)	0.0305 (12)	0.0200 (10)	−0.0064 (10)	0.0053 (9)	−0.0053 (9)
O12	0.0214 (10)	0.0321 (12)	0.0227 (9)	−0.0006 (9)	0.0007 (8)	−0.0057 (9)
O13	0.0392 (11)	0.0301 (12)	0.0187 (10)	0.0061 (10)	0.0107 (8)	0.0021 (9)
O14	0.0340 (11)	0.0280 (11)	0.0174 (9)	0.0039 (9)	0.0114 (8)	−0.0004 (9)
O15	0.0417 (12)	0.0252 (11)	0.0183 (10)	−0.0016 (9)	0.0100 (9)	−0.0022 (9)
O16	0.0284 (10)	0.0247 (11)	0.0224 (9)	0.0050 (9)	0.0043 (8)	−0.0034 (9)
N1	0.0279 (12)	0.0220 (13)	0.0128 (10)	−0.0008 (11)	0.0041 (9)	0.0032 (10)
N2	0.0219 (11)	0.0211 (13)	0.0124 (10)	−0.0002 (10)	0.0016 (9)	0.0001 (9)
N3	0.0202 (11)	0.0273 (13)	0.0129 (10)	0.0012 (10)	0.0053 (9)	−0.0022 (10)
N4	0.0274 (12)	0.0242 (13)	0.0124 (10)	0.0033 (11)	0.0056 (9)	0.0011 (10)
C1	0.0211 (13)	0.0244 (16)	0.0201 (13)	−0.0031 (12)	0.0023 (11)	−0.0013 (12)
C2	0.0275 (14)	0.0249 (16)	0.0170 (13)	−0.0089 (13)	0.0013 (11)	−0.0014 (12)
C3	0.0430 (17)	0.0273 (17)	0.0155 (13)	−0.0111 (14)	−0.0012 (12)	0.0001 (12)
C4	0.0364 (16)	0.0269 (17)	0.0207 (13)	−0.0094 (14)	−0.0024 (12)	0.0055 (13)
C5	0.0282 (15)	0.0204 (16)	0.0329 (15)	−0.0022 (13)	−0.0053 (12)	0.0077 (14)
C6	0.0230 (14)	0.0235 (16)	0.0283 (15)	−0.0025 (13)	0.0011 (12)	0.0002 (13)
C7	0.0204 (14)	0.0270 (16)	0.0175 (13)	−0.0063 (13)	−0.0029 (11)	−0.0005 (12)
C8	0.0300 (15)	0.0215 (16)	0.0210 (14)	−0.0070 (13)	−0.0004 (12)	0.0009 (12)
C9	0.0207 (13)	0.0218 (16)	0.0231 (14)	−0.0048 (12)	−0.0014 (11)	0.0042 (12)
C10	0.0296 (15)	0.0303 (18)	0.0243 (14)	0.0047 (14)	0.0034 (12)	−0.0012 (13)
C11	0.0288 (15)	0.0321 (18)	0.0190 (14)	−0.0094 (14)	0.0048 (12)	0.0005 (13)
C14	0.0210 (13)	0.0192 (15)	0.0188 (13)	0.0006 (12)	0.0059 (11)	0.0020 (12)
C15	0.0232 (14)	0.0164 (14)	0.0156 (12)	0.0027 (12)	0.0031 (10)	0.0014 (11)
C16	0.0243 (14)	0.0139 (14)	0.0173 (13)	0.0047 (12)	0.0039 (11)	0.0031 (11)
C17	0.0303 (15)	0.0171 (15)	0.0216 (13)	0.0011 (12)	0.0117 (12)	−0.0001 (12)
C18	0.0288 (15)	0.0217 (16)	0.0290 (15)	−0.0005 (13)	0.0147 (12)	0.0042 (13)
C19	0.0208 (14)	0.0169 (15)	0.0315 (15)	−0.0006 (12)	0.0073 (12)	0.0015 (12)
C20	0.0240 (14)	0.0170 (15)	0.0214 (14)	−0.0001 (12)	0.0024 (11)	−0.0006 (12)
C21	0.0259 (14)	0.0130 (14)	0.0196 (13)	0.0029 (12)	0.0060 (11)	0.0040 (11)
C22	0.0228 (14)	0.0158 (14)	0.0182 (13)	0.0010 (11)	0.0057 (11)	0.0020 (11)
C23	0.0236 (14)	0.0335 (18)	0.0267 (15)	−0.0030 (14)	−0.0034 (12)	−0.0003 (14)
C24	0.0264 (14)	0.0150 (14)	0.0177 (13)	0.0022 (12)	0.0036 (11)	0.0015 (12)
C25	0.0202 (14)	0.0297 (17)	0.0324 (15)	−0.0038 (13)	−0.0011 (12)	0.0007 (14)
C26	0.0252 (16)	0.067 (3)	0.0371 (17)	−0.0069 (16)	0.0110 (13)	−0.0095 (17)
C27	0.0209 (13)	0.0192 (15)	0.0179 (12)	0.0023 (12)	0.0038 (11)	−0.0009 (12)
C28	0.0254 (14)	0.0167 (14)	0.0153 (12)	0.0015 (12)	0.0043 (11)	0.0015 (11)
C29	0.0305 (15)	0.0189 (15)	0.0144 (13)	0.0053 (12)	0.0068 (11)	0.0035 (12)
C30	0.0302 (15)	0.0247 (16)	0.0249 (14)	0.0050 (13)	0.0132 (12)	0.0052 (13)
C31	0.0237 (14)	0.0267 (17)	0.0386 (16)	0.0020 (13)	0.0161 (13)	0.0072 (14)
C32	0.0210 (14)	0.0168 (15)	0.0341 (15)	0.0027 (12)	0.0033 (12)	0.0035 (13)
C33	0.0257 (14)	0.0185 (15)	0.0225 (13)	0.0025 (12)	0.0068 (11)	−0.0014 (12)
C34	0.0256 (14)	0.0165 (14)	0.0199 (13)	0.0042 (12)	0.0085 (11)	0.0028 (11)
C35	0.0202 (13)	0.0147 (14)	0.0220 (13)	0.0015 (11)	0.0072 (11)	0.0006 (11)
C36	0.0236 (15)	0.061 (3)	0.0285 (15)	−0.0040 (16)	−0.0045 (12)	−0.0112 (16)
C37	0.0314 (15)	0.0163 (15)	0.0159 (13)	0.0025 (12)	0.0020 (11)	0.0025 (12)
C38	0.0236 (14)	0.0288 (18)	0.0349 (16)	−0.0007 (13)	−0.0048 (12)	−0.0047 (14)
C39	0.0274 (15)	0.044 (2)	0.0213 (14)	−0.0011 (15)	0.0005 (12)	−0.0048 (14)
C40	0.0227 (14)	0.0237 (16)	0.0187 (13)	−0.0002 (12)	0.0045 (11)	0.0009 (12)
C41	0.0182 (13)	0.0220 (15)	0.0178 (13)	−0.0003 (12)	0.0038 (10)	0.0017 (12)
C42	0.0212 (13)	0.0261 (16)	0.0145 (13)	−0.0054 (12)	0.0064 (10)	−0.0013 (12)
C43	0.0236 (14)	0.0253 (16)	0.0186 (13)	−0.0015 (13)	0.0045 (11)	0.0042 (12)
C44	0.0236 (14)	0.0205 (16)	0.0300 (15)	0.0007 (12)	0.0061 (12)	0.0040 (13)
C45	0.0244 (14)	0.0201 (16)	0.0265 (14)	0.0001 (13)	0.0086 (12)	−0.0009 (12)
C46	0.0199 (13)	0.0237 (15)	0.0163 (12)	−0.0032 (12)	0.0049 (11)	−0.0010 (12)
C47	0.0180 (13)	0.0206 (15)	0.0190 (13)	−0.0043 (12)	0.0053 (10)	0.0012 (12)
C48	0.0143 (12)	0.0216 (15)	0.0192 (13)	−0.0018 (12)	0.0020 (10)	0.0001 (12)
C49	0.0414 (17)	0.0308 (18)	0.0238 (14)	0.0018 (14)	0.0163 (13)	−0.0062 (13)
C50	0.0197 (13)	0.0242 (16)	0.0185 (13)	−0.0029 (12)	0.0045 (11)	0.0022 (12)
C51	0.0313 (15)	0.0229 (16)	0.0319 (15)	0.0039 (13)	0.0129 (13)	−0.0068 (13)
C52	0.0313 (16)	0.0343 (19)	0.0475 (18)	0.0117 (15)	0.0043 (14)	−0.0051 (16)
C12A	0.029 (11)	0.054 (13)	0.053 (9)	−0.012 (8)	0.020 (9)	−0.040 (9)
C12B	0.039 (5)	0.046 (5)	0.025 (3)	0.005 (4)	0.011 (4)	−0.006 (4)
C13B	0.035 (3)	0.044 (4)	0.028 (3)	0.007 (3)	0.012 (2)	−0.008 (3)
C13A	0.069 (12)	0.089 (15)	0.024 (7)	0.041 (11)	0.024 (7)	0.009 (8)

### Geometric parameters (Å, º)

**Table:**

O1—C3	1.236 (4)	C42—C47	1.473 (4)
O2—C7	1.373 (3)	C43—C44	1.361 (4)
O2—C10	1.428 (4)	C43—C47	1.407 (4)
O3—C11	1.212 (4)	C44—C45	1.404 (4)
O4—C11	1.344 (4)	C45—C46	1.367 (4)
O4—C12A	1.52 (3)	C46—C48	1.410 (4)
O4—C12B	1.466 (11)	C47—C48	1.400 (4)
O5—C16	1.247 (3)	C51—C52	1.501 (4)
O6—C20	1.373 (3)	C12A—C13A	1.57 (3)
O6—C23	1.429 (3)	C12B—C13B	1.465 (12)
O7—C24	1.205 (3)	N1—H1A	0.880
O8—C24	1.353 (4)	N2—H2	0.880
O8—C25	1.458 (3)	N3—H3	0.880
O9—C29	1.249 (3)	N4—H4A	0.880
O10—C33	1.364 (4)	C1—H1	0.950
O10—C36	1.434 (3)	C4—H4	0.950
O11—C37	1.214 (3)	C5—H5	0.950
O12—C37	1.347 (4)	C6—H6	0.950
O12—C38	1.455 (3)	C10—H10A	0.980
O13—C42	1.248 (3)	C10—H10B	0.980
O14—C46	1.369 (3)	C10—H10C	0.980
O14—C49	1.435 (4)	C14—H14	0.950
O15—C50	1.215 (3)	C17—H17	0.950
O16—C50	1.350 (3)	C18—H18	0.950
O16—C51	1.452 (4)	C19—H19	0.950
N1—C1	1.335 (4)	C23—H23A	0.980
N1—C9	1.385 (4)	C23—H23B	0.980
N2—C14	1.330 (3)	C23—H23C	0.980
N2—C22	1.383 (4)	C25—H25A	0.990
N3—C27	1.322 (3)	C25—H25B	0.990
N3—C35	1.384 (4)	C26—H26A	0.980
N4—C40	1.313 (4)	C26—H26B	0.980
N4—C48	1.385 (4)	C26—H26C	0.980
C1—C2	1.381 (4)	C27—H27	0.950
C2—C3	1.451 (4)	C30—H30	0.950
C2—C11	1.468 (4)	C31—H31	0.950
C3—C8	1.484 (4)	C32—H32	0.950
C4—C5	1.362 (4)	C36—H36A	0.980
C4—C8	1.401 (4)	C36—H36B	0.980
C5—C6	1.410 (4)	C36—H36C	0.980
C6—C7	1.368 (4)	C38—H38A	0.990
C7—C9	1.412 (4)	C38—H38B	0.990
C8—C9	1.398 (4)	C39—H39A	0.980
C14—C15	1.375 (4)	C39—H39B	0.980
C15—C16	1.454 (4)	C39—H39C	0.980
C15—C24	1.479 (4)	C40—H40	0.950
C16—C21	1.472 (4)	C43—H43	0.950
C17—C18	1.366 (4)	C44—H44	0.950
C17—C21	1.411 (4)	C45—H45	0.950
C18—C19	1.403 (4)	C49—H49A	0.980
C19—C20	1.371 (4)	C49—H49B	0.980
C20—C22	1.405 (4)	C49—H49C	0.980
C21—C22	1.392 (4)	C51—H51A	0.990
C25—C26	1.503 (5)	C51—H51B	0.990
C27—C28	1.378 (4)	C52—H52A	0.980
C28—C29	1.446 (4)	C52—H52B	0.980
C28—C37	1.469 (4)	C52—H52C	0.980
C29—C34	1.470 (4)	C12A—H12A	0.990
C30—C31	1.367 (4)	C12A—H12B	0.990
C30—C34	1.404 (4)	C12B—H12C	0.990
C31—C32	1.394 (4)	C12B—H12D	0.990
C32—C33	1.381 (4)	C13B—H13D	0.980
C33—C35	1.410 (4)	C13B—H13E	0.980
C34—C35	1.398 (4)	C13B—H13F	0.980
C38—C39	1.493 (4)	C13A—H13A	0.980
C40—C41	1.381 (4)	C13A—H13B	0.980
C41—C42	1.445 (4)	C13A—H13C	0.980
C41—C50	1.471 (4)		
			
C7—O2—C10	116.8 (2)	C48—N4—H4A	119.180
C11—O4—C12A	118.5 (10)	N1—C1—H1	118.465
C11—O4—C12B	112.9 (5)	C2—C1—H1	118.468
C20—O6—C23	117.1 (2)	C5—C4—H4	119.570
C24—O8—C25	116.95 (19)	C8—C4—H4	119.579
C33—O10—C36	115.8 (2)	C4—C5—H5	119.522
C37—O12—C38	117.7 (2)	C6—C5—H5	119.501
C46—O14—C49	116.9 (2)	C5—C6—H6	120.434
C50—O16—C51	114.97 (18)	C7—C6—H6	120.439
C1—N1—C9	121.4 (3)	O2—C10—H10A	109.449
C14—N2—C22	121.6 (2)	O2—C10—H10B	109.463
C27—N3—C35	121.3 (2)	O2—C10—H10C	109.472
C40—N4—C48	121.6 (3)	H10A—C10—H10B	109.476
N1—C1—C2	123.1 (3)	H10A—C10—H10C	109.482
C1—C2—C3	120.1 (3)	H10B—C10—H10C	109.485
C1—C2—C11	119.0 (3)	N2—C14—H14	118.396
C3—C2—C11	121.0 (3)	C15—C14—H14	118.373
O1—C3—C2	124.8 (3)	C18—C17—H17	119.749
O1—C3—C8	120.3 (3)	C21—C17—H17	119.763
C2—C3—C8	114.9 (3)	C17—C18—H18	119.704
C5—C4—C8	120.9 (3)	C19—C18—H18	119.692
C4—C5—C6	121.0 (3)	C18—C19—H19	120.039
C5—C6—C7	119.1 (3)	C20—C19—H19	120.048
O2—C7—C6	125.7 (3)	O6—C23—H23A	109.461
O2—C7—C9	114.1 (3)	O6—C23—H23B	109.472
C6—C7—C9	120.3 (3)	O6—C23—H23C	109.468
C3—C8—C4	120.9 (3)	H23A—C23—H23B	109.467
C3—C8—C9	120.6 (3)	H23A—C23—H23C	109.478
C4—C8—C9	118.4 (3)	H23B—C23—H23C	109.481
N1—C9—C7	120.0 (3)	O8—C25—H25A	110.428
N1—C9—C8	119.7 (3)	O8—C25—H25B	110.435
C7—C9—C8	120.3 (3)	C26—C25—H25A	110.441
O3—C11—O4	121.9 (3)	C26—C25—H25B	110.440
O3—C11—C2	125.3 (3)	H25A—C25—H25B	108.637
O4—C11—C2	112.9 (3)	C25—C26—H26A	109.481
N2—C14—C15	123.2 (3)	C25—C26—H26B	109.475
C14—C15—C16	119.7 (2)	C25—C26—H26C	109.464
C14—C15—C24	119.1 (3)	H26A—C26—H26B	109.474
C16—C15—C24	121.3 (2)	H26A—C26—H26C	109.462
O5—C16—C15	124.8 (3)	H26B—C26—H26C	109.471
O5—C16—C21	120.2 (3)	N3—C27—H27	118.144
C15—C16—C21	115.0 (2)	C28—C27—H27	118.141
C18—C17—C21	120.5 (3)	C31—C30—H30	119.924
C17—C18—C19	120.6 (3)	C34—C30—H30	119.929
C18—C19—C20	119.9 (3)	C30—C31—H31	119.357
O6—C20—C19	126.4 (2)	C32—C31—H31	119.367
O6—C20—C22	113.9 (3)	C31—C32—H32	120.064
C19—C20—C22	119.7 (3)	C33—C32—H32	120.076
C16—C21—C17	120.5 (3)	O10—C36—H36A	109.470
C16—C21—C22	121.1 (3)	O10—C36—H36B	109.468
C17—C21—C22	118.4 (3)	O10—C36—H36C	109.477
N2—C22—C20	120.1 (2)	H36A—C36—H36B	109.463
N2—C22—C21	119.2 (2)	H36A—C36—H36C	109.482
C20—C22—C21	120.7 (3)	H36B—C36—H36C	109.467
O7—C24—O8	122.4 (3)	O12—C38—H38A	110.084
O7—C24—C15	126.8 (3)	O12—C38—H38B	110.079
O8—C24—C15	110.8 (2)	C39—C38—H38A	110.071
O8—C25—C26	106.5 (2)	C39—C38—H38B	110.075
N3—C27—C28	123.7 (3)	H38A—C38—H38B	108.416
C27—C28—C29	119.3 (2)	C38—C39—H39A	109.465
C27—C28—C37	119.5 (3)	C38—C39—H39B	109.469
C29—C28—C37	121.2 (3)	C38—C39—H39C	109.467
O9—C29—C28	124.3 (3)	H39A—C39—H39B	109.476
O9—C29—C34	119.9 (3)	H39A—C39—H39C	109.467
C28—C29—C34	115.7 (2)	H39B—C39—H39C	109.483
C31—C30—C34	120.1 (3)	N4—C40—H40	118.233
C30—C31—C32	121.3 (3)	C41—C40—H40	118.243
C31—C32—C33	119.9 (3)	C44—C43—H43	119.611
O10—C33—C32	125.4 (3)	C47—C43—H43	119.613
O10—C33—C35	115.3 (3)	C43—C44—H44	119.518
C32—C33—C35	119.3 (3)	C45—C44—H44	119.533
C29—C34—C30	120.8 (3)	C44—C45—H45	120.107
C29—C34—C35	120.3 (3)	C46—C45—H45	120.099
C30—C34—C35	118.8 (2)	O14—C49—H49A	109.459
N3—C35—C33	119.9 (3)	O14—C49—H49B	109.467
N3—C35—C34	119.6 (2)	O14—C49—H49C	109.474
C33—C35—C34	120.5 (3)	H49A—C49—H49B	109.475
O11—C37—O12	122.8 (3)	H49A—C49—H49C	109.477
O11—C37—C28	125.5 (3)	H49B—C49—H49C	109.475
O12—C37—C28	111.7 (2)	O16—C51—H51A	110.256
O12—C38—C39	108.1 (3)	O16—C51—H51B	110.268
N4—C40—C41	123.5 (3)	C52—C51—H51A	110.255
C40—C41—C42	119.5 (3)	C52—C51—H51B	110.268
C40—C41—C50	119.5 (3)	H51A—C51—H51B	108.515
C42—C41—C50	120.9 (2)	C51—C52—H52A	109.468
O13—C42—C41	124.5 (3)	C51—C52—H52B	109.471
O13—C42—C47	119.9 (3)	C51—C52—H52C	109.462
C41—C42—C47	115.6 (2)	H52A—C52—H52B	109.481
C44—C43—C47	120.8 (3)	H52A—C52—H52C	109.468
C43—C44—C45	120.9 (3)	H52B—C52—H52C	109.478
C44—C45—C46	119.8 (3)	O4—C12A—H12A	111.542
O14—C46—C45	126.5 (3)	O4—C12A—H12B	111.524
O14—C46—C48	113.9 (3)	C13A—C12A—H12A	111.524
C45—C46—C48	119.6 (3)	C13A—C12A—H12B	111.522
C42—C47—C43	121.8 (3)	H12A—C12A—H12B	109.344
C42—C47—C48	120.2 (3)	O4—C12B—H12C	109.639
C43—C47—C48	118.0 (3)	O4—C12B—H12D	109.628
N4—C48—C46	119.8 (3)	C13B—C12B—H12C	109.631
N4—C48—C47	119.4 (3)	C13B—C12B—H12D	109.629
C46—C48—C47	120.8 (3)	H12C—C12B—H12D	108.141
O15—C50—O16	122.1 (3)	C12B—C13B—H13D	109.464
O15—C50—C41	125.2 (3)	C12B—C13B—H13E	109.486
O16—C50—C41	112.6 (2)	C12B—C13B—H13F	109.469
O16—C51—C52	107.3 (3)	H13D—C13B—H13E	109.471
O4—C12A—C13A	101.2 (15)	H13D—C13B—H13F	109.469
O4—C12B—C13B	110.1 (7)	H13E—C13B—H13F	109.469
C1—N1—H1A	119.319	C12A—C13A—H13A	109.463
C9—N1—H1A	119.326	C12A—C13A—H13B	109.488
C14—N2—H2	119.188	C12A—C13A—H13C	109.480
C22—N2—H2	119.186	H13A—C13A—H13B	109.471
C27—N3—H3	119.376	H13A—C13A—H13C	109.455
C35—N3—H3	119.367	H13B—C13A—H13C	109.470
C40—N4—H4A	119.184		
			
C7—O2—C10—H10A	60.2	H17—C17—C18—H18	−2.7
C7—O2—C10—H10B	−59.8	H17—C17—C21—C16	2.2
C7—O2—C10—H10C	−179.8	H17—C17—C21—C22	−179.1
C10—O2—C7—C6	−3.6 (4)	C17—C18—C19—C20	1.4 (4)
C10—O2—C7—C9	178.04 (18)	C17—C18—C19—H19	−178.6
C11—O4—C12A—C13A	−112.4 (11)	H18—C18—C19—C20	−178.6
C11—O4—C12A—H12A	128.9	H18—C18—C19—H19	1.4
C11—O4—C12A—H12B	6.3	C18—C19—C20—O6	−179.2 (3)
C12A—O4—C11—O3	17.5 (10)	C18—C19—C20—C22	1.5 (4)
C12A—O4—C11—C2	−164.0 (10)	H19—C19—C20—O6	0.8
C11—O4—C12B—C13B	−167.1 (4)	H19—C19—C20—C22	−178.5
C11—O4—C12B—H12C	−46.4	O6—C20—C22—N2	−3.9 (4)
C11—O4—C12B—H12D	72.2	O6—C20—C22—C21	177.35 (19)
C12B—O4—C11—O3	−6.0 (5)	C19—C20—C22—N2	175.4 (3)
C12B—O4—C11—C2	172.4 (4)	C19—C20—C22—C21	−3.3 (4)
C12A—O4—C12B—C13B	83 (3)	C16—C21—C22—N2	2.0 (4)
C12A—O4—C12B—H12C	−156.1	C16—C21—C22—C20	−179.2 (2)
C12A—O4—C12B—H12D	−37.5	C17—C21—C22—N2	−176.7 (2)
C12B—O4—C12A—C13A	−31.8 (16)	C17—C21—C22—C20	2.1 (4)
C12B—O4—C12A—H12A	−150.5	O8—C25—C26—H26A	55.7
C12B—O4—C12A—H12B	86.9	O8—C25—C26—H26B	−64.3
C20—O6—C23—H23A	177.4	O8—C25—C26—H26C	175.7
C20—O6—C23—H23B	57.4	H25A—C25—C26—H26A	175.6
C20—O6—C23—H23C	−62.6	H25A—C25—C26—H26B	55.6
C23—O6—C20—C19	1.8 (4)	H25A—C25—C26—H26C	−64.4
C23—O6—C20—C22	−178.91 (19)	H25B—C25—C26—H26A	−64.2
C24—O8—C25—C26	157.98 (18)	H25B—C25—C26—H26B	175.8
C24—O8—C25—H25A	38.1	H25B—C25—C26—H26C	55.8
C24—O8—C25—H25B	−82.1	N3—C27—C28—C29	−0.1 (4)
C25—O8—C24—O7	−8.2 (4)	N3—C27—C28—C37	177.2 (2)
C25—O8—C24—C15	171.78 (18)	H27—C27—C28—C29	179.9
C33—O10—C36—H36A	65.9	H27—C27—C28—C37	−2.8
C33—O10—C36—H36B	−54.1	C27—C28—C29—O9	179.5 (3)
C33—O10—C36—H36C	−174.1	C27—C28—C29—C34	−1.9 (4)
C36—O10—C33—C32	−4.6 (4)	C27—C28—C37—O11	−158.9 (3)
C36—O10—C33—C35	175.1 (2)	C27—C28—C37—O12	19.8 (4)
C37—O12—C38—C39	−148.8 (2)	C29—C28—C37—O11	18.4 (4)
C37—O12—C38—H38A	90.9	C29—C28—C37—O12	−162.9 (2)
C37—O12—C38—H38B	−28.6	C37—C28—C29—O9	2.2 (4)
C38—O12—C37—O11	3.7 (4)	C37—C28—C29—C34	−179.2 (2)
C38—O12—C37—C28	−175.01 (19)	O9—C29—C34—C30	1.0 (4)
C46—O14—C49—H49A	59.8	O9—C29—C34—C35	−178.7 (2)
C46—O14—C49—H49B	−60.2	C28—C29—C34—C30	−177.6 (2)
C46—O14—C49—H49C	179.8	C28—C29—C34—C35	2.7 (4)
C49—O14—C46—C45	7.3 (4)	C31—C30—C34—C29	−179.8 (3)
C49—O14—C46—C48	−172.14 (18)	C31—C30—C34—C35	−0.2 (4)
C50—O16—C51—C52	−175.17 (18)	C34—C30—C31—C32	1.0 (4)
C50—O16—C51—H51A	64.7	C34—C30—C31—H31	−179.0
C50—O16—C51—H51B	−55.1	H30—C30—C31—C32	−179.0
C51—O16—C50—O15	0.5 (4)	H30—C30—C31—H31	1.0
C51—O16—C50—C41	−179.79 (18)	H30—C30—C34—C29	0.2
C1—N1—C9—C7	174.2 (2)	H30—C30—C34—C35	179.8
C1—N1—C9—C8	−4.3 (4)	C30—C31—C32—C33	0.1 (4)
C9—N1—C1—C2	1.0 (4)	C30—C31—C32—H32	−179.9
C9—N1—C1—H1	−179.0	H31—C31—C32—C33	−179.9
H1A—N1—C1—C2	−179.0	H31—C31—C32—H32	0.1
H1A—N1—C1—H1	1.0	C31—C32—C33—O10	177.6 (3)
H1A—N1—C9—C7	−5.8	C31—C32—C33—C35	−2.0 (4)
H1A—N1—C9—C8	175.8	H32—C32—C33—O10	−2.4
C14—N2—C22—C20	−176.7 (2)	H32—C32—C33—C35	178.0
C14—N2—C22—C21	2.1 (4)	O10—C33—C35—N3	2.6 (4)
C22—N2—C14—C15	−3.4 (4)	O10—C33—C35—C34	−176.83 (19)
C22—N2—C14—H14	176.6	C32—C33—C35—N3	−177.7 (2)
H2—N2—C14—C15	176.6	C32—C33—C35—C34	2.8 (4)
H2—N2—C14—H14	−3.4	C29—C34—C35—N3	−1.5 (4)
H2—N2—C22—C20	3.3	C29—C34—C35—C33	177.9 (2)
H2—N2—C22—C21	−177.9	C30—C34—C35—N3	178.8 (2)
C27—N3—C35—C33	179.9 (2)	C30—C34—C35—C33	−1.7 (4)
C27—N3—C35—C34	−0.7 (4)	O12—C38—C39—H39A	−52.5
C35—N3—C27—C28	1.5 (4)	O12—C38—C39—H39B	−172.5
C35—N3—C27—H27	−178.5	O12—C38—C39—H39C	67.4
H3—N3—C27—C28	−178.5	H38A—C38—C39—H39A	67.7
H3—N3—C27—H27	1.5	H38A—C38—C39—H39B	−52.3
H3—N3—C35—C33	−0.1	H38A—C38—C39—H39C	−172.3
H3—N3—C35—C34	179.3	H38B—C38—C39—H39A	−172.8
C40—N4—C48—C46	178.00 (19)	H38B—C38—C39—H39B	67.2
C40—N4—C48—C47	−1.2 (4)	H38B—C38—C39—H39C	−52.8
C48—N4—C40—C41	−1.6 (4)	N4—C40—C41—C42	3.7 (4)
C48—N4—C40—H40	178.4	N4—C40—C41—C50	−175.2 (2)
H4A—N4—C40—C41	178.4	H40—C40—C41—C42	−176.3
H4A—N4—C40—H40	−1.6	H40—C40—C41—C50	4.8
H4A—N4—C48—C46	−2.0	C40—C41—C42—O13	175.7 (2)
H4A—N4—C48—C47	178.8	C40—C41—C42—C47	−2.9 (3)
N1—C1—C2—C3	4.3 (4)	C40—C41—C50—O15	161.5 (3)
N1—C1—C2—C11	−174.7 (2)	C40—C41—C50—O16	−18.2 (3)
H1—C1—C2—C3	−175.7	C42—C41—C50—O15	−17.4 (4)
H1—C1—C2—C11	5.3	C42—C41—C50—O16	163.0 (2)
C1—C2—C3—O1	174.9 (3)	C50—C41—C42—O13	−5.4 (4)
C1—C2—C3—C8	−5.9 (4)	C50—C41—C42—C47	175.99 (19)
C1—C2—C11—O3	166.6 (3)	O13—C42—C47—C43	3.0 (4)
C1—C2—C11—O4	−11.7 (4)	O13—C42—C47—C48	−178.39 (19)
C3—C2—C11—O3	−12.4 (4)	C41—C42—C47—C43	−178.36 (19)
C3—C2—C11—O4	169.3 (3)	C41—C42—C47—C48	0.3 (3)
C11—C2—C3—O1	−6.1 (4)	C44—C43—C47—C42	178.1 (2)
C11—C2—C3—C8	173.1 (2)	C44—C43—C47—C48	−0.5 (4)
O1—C3—C8—C4	3.7 (4)	C47—C43—C44—C45	0.5 (4)
O1—C3—C8—C9	−178.0 (3)	C47—C43—C44—H44	−179.5
C2—C3—C8—C4	−175.6 (2)	H43—C43—C44—C45	−179.5
C2—C3—C8—C9	2.8 (4)	H43—C43—C44—H44	0.5
C5—C4—C8—C3	177.7 (3)	H43—C43—C47—C42	−1.9
C5—C4—C8—C9	−0.6 (4)	H43—C43—C47—C48	179.5
C8—C4—C5—C6	−1.6 (4)	C43—C44—C45—C46	−1.1 (4)
C8—C4—C5—H5	178.4	C43—C44—C45—H45	178.8
H4—C4—C5—C6	178.4	H44—C44—C45—C46	178.8
H4—C4—C5—H5	−1.5	H44—C44—C45—H45	−1.2
H4—C4—C8—C3	−2.3	C44—C45—C46—O14	−177.6 (2)
H4—C4—C8—C9	179.3	C44—C45—C46—C48	1.8 (4)
C4—C5—C6—C7	2.3 (4)	H45—C45—C46—O14	2.4
C4—C5—C6—H6	−177.7	H45—C45—C46—C48	−178.2
H5—C5—C6—C7	−177.7	O14—C46—C48—N4	−1.6 (3)
H5—C5—C6—H6	2.3	O14—C46—C48—C47	177.60 (18)
C5—C6—C7—O2	−179.1 (2)	C45—C46—C48—N4	178.9 (2)
C5—C6—C7—C9	−0.8 (4)	C45—C46—C48—C47	−1.9 (4)
H6—C6—C7—O2	0.9	C42—C47—C48—N4	1.8 (4)
H6—C6—C7—C9	179.2	C42—C47—C48—C46	−177.45 (19)
O2—C7—C9—N1	−1.4 (3)	C43—C47—C48—N4	−179.54 (19)
O2—C7—C9—C8	177.04 (18)	C43—C47—C48—C46	1.2 (4)
C6—C7—C9—N1	−179.8 (2)	O16—C51—C52—H52A	62.3
C6—C7—C9—C8	−1.4 (4)	O16—C51—C52—H52B	−57.7
C3—C8—C9—N1	2.2 (4)	O16—C51—C52—H52C	−177.7
C3—C8—C9—C7	−176.2 (2)	H51A—C51—C52—H52A	−177.6
C4—C8—C9—N1	−179.5 (2)	H51A—C51—C52—H52B	62.4
C4—C8—C9—C7	2.1 (4)	H51A—C51—C52—H52C	−57.6
N2—C14—C15—C16	0.4 (4)	H51B—C51—C52—H52A	−57.8
N2—C14—C15—C24	179.6 (2)	H51B—C51—C52—H52B	−177.8
H14—C14—C15—C16	−179.6	H51B—C51—C52—H52C	62.2
H14—C14—C15—C24	−0.4	O4—C12A—C13A—H13A	−61.5
C14—C15—C16—O5	−177.2 (3)	O4—C12A—C13A—H13B	178.5
C14—C15—C16—C21	3.4 (4)	O4—C12A—C13A—H13C	58.5
C14—C15—C24—O7	171.8 (3)	H12A—C12A—C13A—H13A	57.3
C14—C15—C24—O8	−8.1 (4)	H12A—C12A—C13A—H13B	−62.8
C16—C15—C24—O7	−9.0 (4)	H12A—C12A—C13A—H13C	177.2
C16—C15—C24—O8	171.1 (2)	H12B—C12A—C13A—H13A	179.8
C24—C15—C16—O5	3.6 (4)	H12B—C12A—C13A—H13B	59.8
C24—C15—C16—C21	−175.8 (2)	H12B—C12A—C13A—H13C	−60.2
O5—C16—C21—C17	−5.3 (4)	O4—C12B—C13B—H13D	−47.5
O5—C16—C21—C22	176.0 (2)	O4—C12B—C13B—H13E	−167.5
C15—C16—C21—C17	174.1 (2)	O4—C12B—C13B—H13F	72.5
C15—C16—C21—C22	−4.6 (4)	H12C—C12B—C13B—H13D	−168.2
C18—C17—C21—C16	−177.8 (2)	H12C—C12B—C13B—H13E	71.8
C18—C17—C21—C22	0.9 (4)	H12C—C12B—C13B—H13F	−48.2
C21—C17—C18—C19	−2.7 (4)	H12D—C12B—C13B—H13D	73.2
C21—C17—C18—H18	177.3	H12D—C12B—C13B—H13E	−46.8
H17—C17—C18—C19	177.3	H12D—C12B—C13B—H13F	−166.8

### Hydrogen-bond geometry (Å, º)

**Table:**

*D*—H···*A*	*D*—H	H···*A*	*D*···*A*	*D*—H···*A*
N1—H1*A*···O2	0.88	2.33	2.665 (3)	103
N1—H1*A*···O5	0.88	1.93	2.729 (3)	151
N2—H2···O3^i^	0.88	2.19	2.873 (3)	134
N2—H2···O6	0.88	2.31	2.656 (4)	103
N3—H3···O10	0.88	2.35	2.685 (3)	103
N3—H3···O13^ii^	0.88	2.21	2.857 (4)	130
N3—H3···O15^ii^	0.88	2.26	2.898 (3)	129
N4—H4*A*···O9	0.88	2.03	2.716 (3)	134
N4—H4*A*···O14	0.88	2.31	2.652 (3)	103

Symmetry codes: (i) *x*, −*y*−1/2, *z*−1/2; (ii) *x*, −*y*+1/2, *z*+1/2.
